# Management, outcome, and novel classification system of periprosthetic fractures in patients with transcutaneous osseointegrated prosthetic systems (TOPS)—a retrospective cohort analysis

**DOI:** 10.1007/s00402-021-03826-y

**Published:** 2021-03-06

**Authors:** Marcus Örgel, Maximilian Petri, Alexander Ranker, Nils Wirries, Tilman Graulich, Christian Krettek, Marcel Winkelmann, Horst-Heinrich Aschoff

**Affiliations:** 1 Trauma Department, Hannover Medical School (MHH), Carl-Neuberg-Straße 1, 30625 Hannover, Germany; 2 Orthopaedic Department, Diakovere Annastift, Anna-von-Borries-Straße 1-7, 30625 Hannover, Germany; 3 Department of Physical Medicine and Rehabilitation, Hannover Medical School (MHH), Carl-Neuberg-Straße 1, 30625 Hannover, Germany

**Keywords:** Periprosthetic fractures, Transcutaneous osseointegrated prosthetic system (TOPS), Endo-exo-prosthesis, Bone anchored prosthetic systems, Amputation, Rehabilitation

## Abstract

**Introduction:**

Transcutaneous osseointegrated prosthetic systems (TOPS) are anchored prosthetic systems for major limb loss. Sometimes TOPS patients suffer from periprosthetic fractures. The aim of this study was to analyze the management and outcomes of periprosthetic fractures in patients with TOPS and to introduce a novel classification system for this entity.

**Material/methods:**

Since 2010, 140 patients were treated with TOPS after transfemoral amputation in two centers in Germany. Fifteen patients sustained periprosthetic fractures, with five intra- and ten postoperative fractures. The outcome was analyzed by Prosthesis Mobility Questionnaire (PMQ), K-level and prosthesis wear time per day. A subgroup analysis for the body mass index (BMI) was performed.

**Results:**

All postoperative fractures were treated with implant-retaining osteosynthesis. Fourteen fractures healed without complications after a mean of 3 months. One postoperative fracture developed a clinically asymptomatic firm non-union. No Endo-Fixstem had to be removed. For the fracture and control group, a significant increase of the PMQ (*p* < 0.001) and K-level (*p* < 0.001) was observed after TOPS treatment compared to the preoperative baseline. Furthermore, the subgroup analysis showed a significant increase of the PMQ and K-level for both normal weight (*p* = 0.002) and overweight patients (*p* < 0.001). Of interest, overweight patients even showed a significantly higher increase in scores compared to normal weight patients, regardless of periprosthetic fracture.

**Conclusion:**

Periprosthetic fractures do not necessarily worsen outcomes of TOPS treatment. Proper classification and standardized appropriate treatment strategies according to fracture morphology are paramount for reliably good outcomes. We recommend to not remove or exchange the implant (Endo-Fixstem) even if it is assembly. Higher BMI did not have an impact onto rehabilitation success after TOPS to major limb loss of the lower extremity.

## Introduction

In Germany, TOPS have been used for more than 15 years [1–3]. This procedure is applicable for patients suffering from an unsatisfying rehabilitation with socket prostheses due to soft tissue problems, short residual limbs, or other inabilities to fit any kind of socket prosthesis after transfemoral amputation [4, 5]. One of the TOPS models is the endo-exo-prosthesis (EEP).

Endo-exo-prosthesis procedure includes two surgical steps at intervals of 4–6 weeks. In the first step, the Endo-Fixstem (implant) is anchored to the bone (endo). Depending on bone quality and primary stability during the first surgery, a stoma is performed in the second step at least four to six weeks after the first surgery with assembling of the components passing through the skin, to which the prosthetist and orthotist connects—the exo-prosthetics [2, 6]. After the first surgery, the bone grows into the three-dimensional surface structure (tripods) of the Endo-Fixstem and creates a strong connection between the bone and prosthesis. This provides a stable walking ability for the patient [2, 7]. Rehabilitation starts with walking on crutches, parallel bars, or other helping tools.

Mostly, this type of prostheses leads to satisfying rehabilitation results with increasing mobility and daily activities [8, 9]. Leijendekkers et al. showed a significant increase of strength, prosthetic use, walking distance, health-related quality of life (HRQoL), and satisfaction level in their prospective one-year follow-up study [9]. Also, Brånemark et al. showed significant improvements for the use of the prosthesis, better mobility, and HRQoL [8].

A substantial incidence of periprosthetic fractures by falls has to be expected [8–12]. So far, there is only one study available about the risk of periprosthetic fractures in patients with osseointegrated implants after transfemoral amputation [12]. In this cohort, 22 patients suffered from a periprosthetic fracture related to TOPS. Neither the K-level nor the prosthesis wear time was negatively affected after fixation of the fracture in any patient [12].

According to this work, we analyzed periprosthetic fractures according to the management and outcome and described a novel classification system as well as treatment algorithm for periprosthetic fractures after TOPS treatment following transfemoral amputation in our consecutive cohort.

### Objectives

The aims of this retrospective study were:

- To investigate the impact of periprosthetic fractures in patients with TOPS by comparing the outcomes in mobility [Prothesis Mobility Questionnaire (PMQ), K-level] and prosthesis wear time in hours in TOPS patients with a periprosthetic fracture to TOPS patients without a periprosthetic fracture,
- To derive a classification system and treatment algorithm of periprosthetic fractures related to TOPS.

Our hypothesis was that there is no difference in the outcome of mobility (PMQ, K-level) and prosthesis wear time per day (in hours) in the fracture group compared to the non-fracture group.

## Methods

This retrospective observational study examines the outcomes of TOPS patients who suffered from an intra- or postoperative periprosthetic fracture. In addition to demographic data, we assessed the mobility by the “Prothesis Mobility Questionnaire” (PMQ) as well as the K-level and the prosthesis wear time per day. These results were compared with TOPS patients who did not sustain a periprosthetic fracture. In addition, the cohort was divided into two subgroups according to their BMI (BMI < 25 kg/m^2^ “normal weight patients” versus BMI ≥ 25 kg/m^2^ “overweight patients”) for analysis of the above-mentioned parameters.

Data collection was performed from an existing database of the Trauma Department of a University Hospital as well as through a structured telephone interview. Regardless to complications such as a periprosthetic fractures, all TOPS patients were evaluated in our clinic within the scope of a standardized assessment before, 3, 6, and 12 months after implantation of the Endo-Fixstem.

**Fig. 1:**
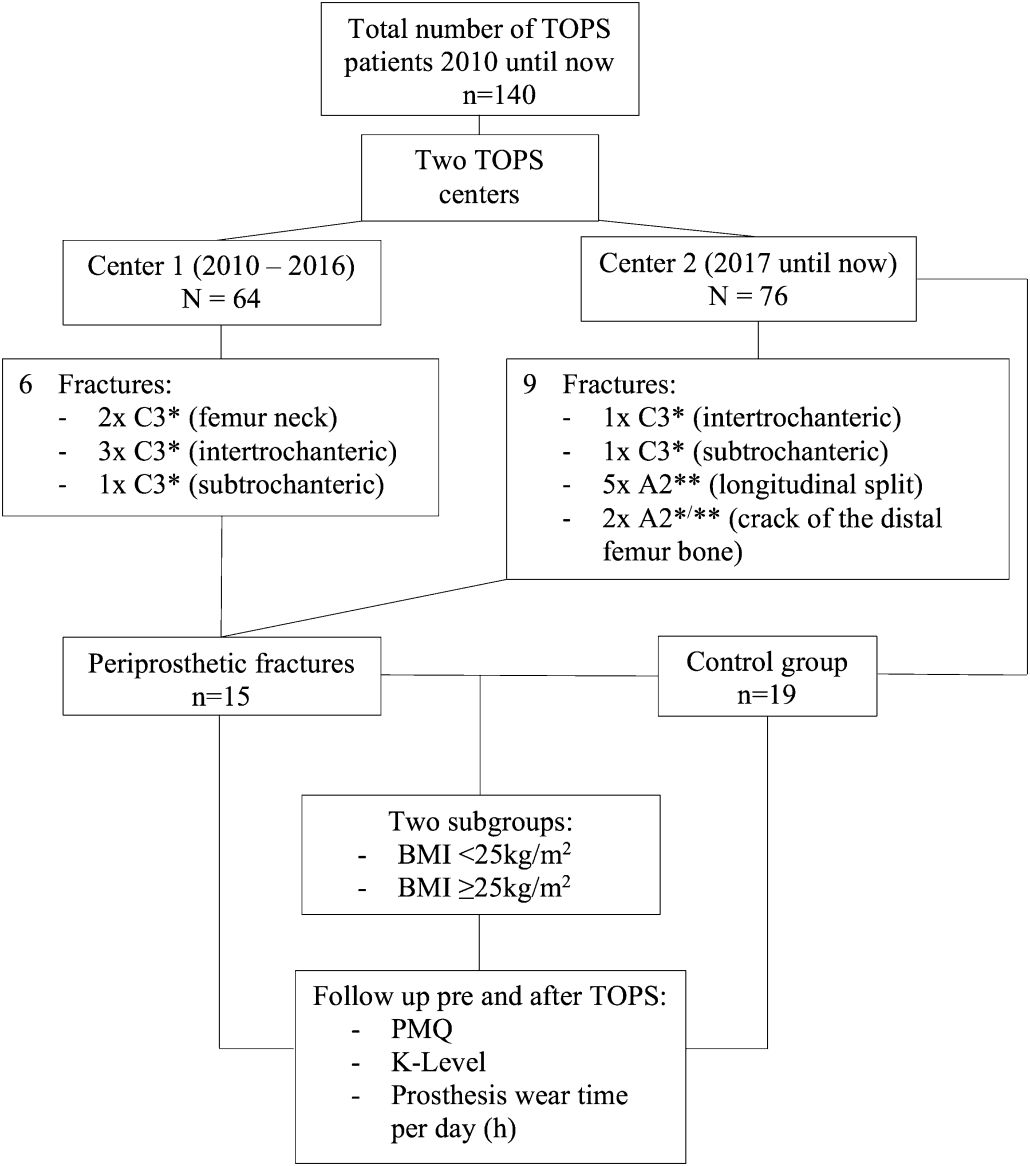
Methods to examine subgroups and interactions; *Hannover Classification of postoperative periprosthetic Fractures (HCpopF); **Hannover Classification of intraoperative periprosthetic Fractures (HCiopF);

Between 2010 and 2017, 64 consecutive patients were included from center 1, while between 2017 and 2019, 76 consecutive patients were included from center 2 (Fig. 1). The reason for recruitment from two centers was professional relocation of the senior surgeon in 2017.

We included several demographic and clinical variables. Patient demographics included age and gender as well as age at amputation, time between amputation to TOPS, and sustaining the fracture. Clinical variables included BMI, PMQ, and K-level as well as time of using the prosthesis per day. Comorbidity was measured with Charlson Comorbidity Index (CCI) [13–15] and peri-/postoperative risk assed with American Society of Anesthesiologists (ASA) [16]. Detailed results are shown in Tables 1 and 2.

**Table 1:** Demographic data of the whole cohort within subgroups (fracture and control group)

	Total (*n* = 34)	Fracture group (*n* = 15)	Control group (*n* = 19)	*p* value
Sex, *n* (%)				0.2^a^
Male	25 (73.5)	13 (86.7)	12 (63.2)	
Female	9 (26.5)	2 (13.3)	7 (36.8)	
Side, *n* (%)				0.3^d^
Left	13 (38.2)	8 (53.3)	11 (57.9)	
Right	19 (55.9)	5 (33.3)	0 (0)	
Bilateral	2 (5.9)	2 (13.3)	8 (42.1)	
Reason for amputation, *n* (%)				0.5^d^
Trauma	23 (67.6)	12 (80.0)	11 (57.9)	
Tumor	1 (2.9)	0	1 (5.3)	
Vascular disease	1 (2.9)	0	1 (5.3)	
Iatrogenic complications	9 (26.5)	3 (20.0)	6 (31.6)	
Age [years], mean ± SD (95%-CI)	48.7 ± 9.6 (45.4–52.1)	49.1 ± 11.6 (42.7–55.6)	48.4 ± 8.1 (44.5–52.3)	0.8^e^
Age at amputation [years] mean ± SD (95%-CI)	32.9 ± 13.0 (28.5–37.5)	29.9 ± 13.3 (22.5–37.2)	35.4 ± 12.5 (29.4–41.5)	0.2^b^
BMI [kg/m^2^] mean ± SD (95%-CI)	26.6 ± 4.3 (25.1–28.1)	25.7 ± 4.5 (23.2–28.2)	27.3 ± 4.2 (25.3–29.4)	0.3^e^
Months amputation to TOPS mean ± SD (95%-CI)	149.7 ± 132.6 (103.4–196.1)	167.5 ± 150.2 (84.3–250.7)	135.7 ± 119.3 (78.2–193.2)	0.6^b^
Prosthesis wear time per day [hours] mean ± SD (95%-CI)	12.8 ± 4.0 (11.4–14.2)	12.1 ± 4.2 (9.8–14.5)	13.3 ± 3.9 (11.4–15.1)	0.2^b^
ASA Score mean ± SD (95%-CI)	2.0 ± 0.3 (1.9–2.1)	1.9 ± 0.5 (1.7–2.2)	2.1 ± 0.2 (1.9–2.2)	0.3^d^
CCI [%] mean ± SD (95%-CI)	94.3 ± 8.8 (91.2–97.4)	92.5 ± 12.2 (85.8–99.3)	95.7 ± 4.6 (93.5–98.0)	0.7^b^

**Table 2:** Data of special findings to the fracture group

	Fracture group (*n* = 15)
Cause of fracture, *n* (%)	
Slipped	2 (13.3)
Stumbling	5 (33.3)
Malfunction of the prosthesis	1 (6.7)
Intraoperative fracture	7 (46.7)
Morphology of the fractures, *n* (%)	
C3*** (femur neck)	2 (13.3)
C3*** (intertrochanteric)	4 (26.7)
C3*** (subtrochanteric)	2 (13.3)
A2*** (longitudinal split of the femur)	5 (33.3)
A2*** (distal femur)	2 (13.3)
Treatment, *n* (%) DCS* plate 95° + cerclage wire LISS** plate + cerclage wire Condylar plate 95° + cerclage wire Only cable wire Individual implant Dynamic hip screw Non-operative	1 (6.7) 2 (13.3) 1 (6.7) 1 (6.7) 1 (6.7) 2 (13.3) 7 (46.7) 8 (53.3)
Time to osteosynthesis [days] mean ± SD (95%-CI)	2.0 ± 1.6 (0.6–3.3)
TOPS to fracture [months] mean ± SD (95%-CI)	21.8 ± 37.8 (-9.8–53.3)

*DCS: dynamic condyle screw; **Less invasive stabilization system; ***Hannover Classification of postoperative periprosthetic Fractures (HCpopF)

The original PMQ is a questionnaire with 12 questions about mobility in everyday life, which are answered on a 5-step Likert scale [17]. The PMQ 2.0 used in this study was derived from first version. The PMQ 2.0 is based on a Rasch analysis by Burger et al., which showed that in case of conflicting questions (e.g., I find it difficult to go upstairs or downstairs), it seems to make more sense to include only those questions in the overall score that are associated with greater difficulties [18]. The maximum total score is 40, which presents the highest score for mobility.

In 1995, the US Health Care Financing Administration (HCFA) [19], a public administration and monitoring agency of the US Medicare and Medicaid program, introduced a classification system (K-level) for leg amputees. It consists of five function levels; K0 ("nonambulator") to K4 ("high-lever user"), which are based on the abilities and potential of an amputee [20]. The classification refers to the walking potential of a patient and is based on subjective patient surveys [21]. Furthermore, the prosthesis wear time was documented in hours per day, which could indirectly provide an indication of mobility and prosthesis satisfaction.

In order to achieve the highest possible degree of objective comparability of the control group, we based the formation of the comparison cohort on parameters of the fracture group, such as gender, age, BMI, and the time of amputation and TOPS care and randomly selected patients from our database who had comparable basic data.

The study size was based on the number of all patients treated with TOPS in Germany since 2010 who suffered a fracture (*n* = 15) and were presented to our clinic. The comparison group (*n* = 19) was randomly selected from the existing database of TOPS patients.

Statistical analysis was performed using SPSS 26 (IBM, SPSS Inc., Chicago, IL). After checking for normal distribution Student’s *t* test^e^ was used for normal and Mann–Whitney *U* test^b^ as well as Wilcoxon test^c^ for non-normal variables. Fisher’s exact^a^ test and Pearson’s Chi-squared test^d^ were used for categorial variables. Significance was set to *p* < 0.05.

## Results

Follow-up data could be completely obtained from all patients (*n* = 15). Comorbidity was measured with Charlson Comorbidity Index (CCI) [13–15] and peri-/postoperative risk assed with American Society of Anesthesiologists (ASA) [16]. Detailed results are shown in Tables 1 and 2.

**Fig. 2:**
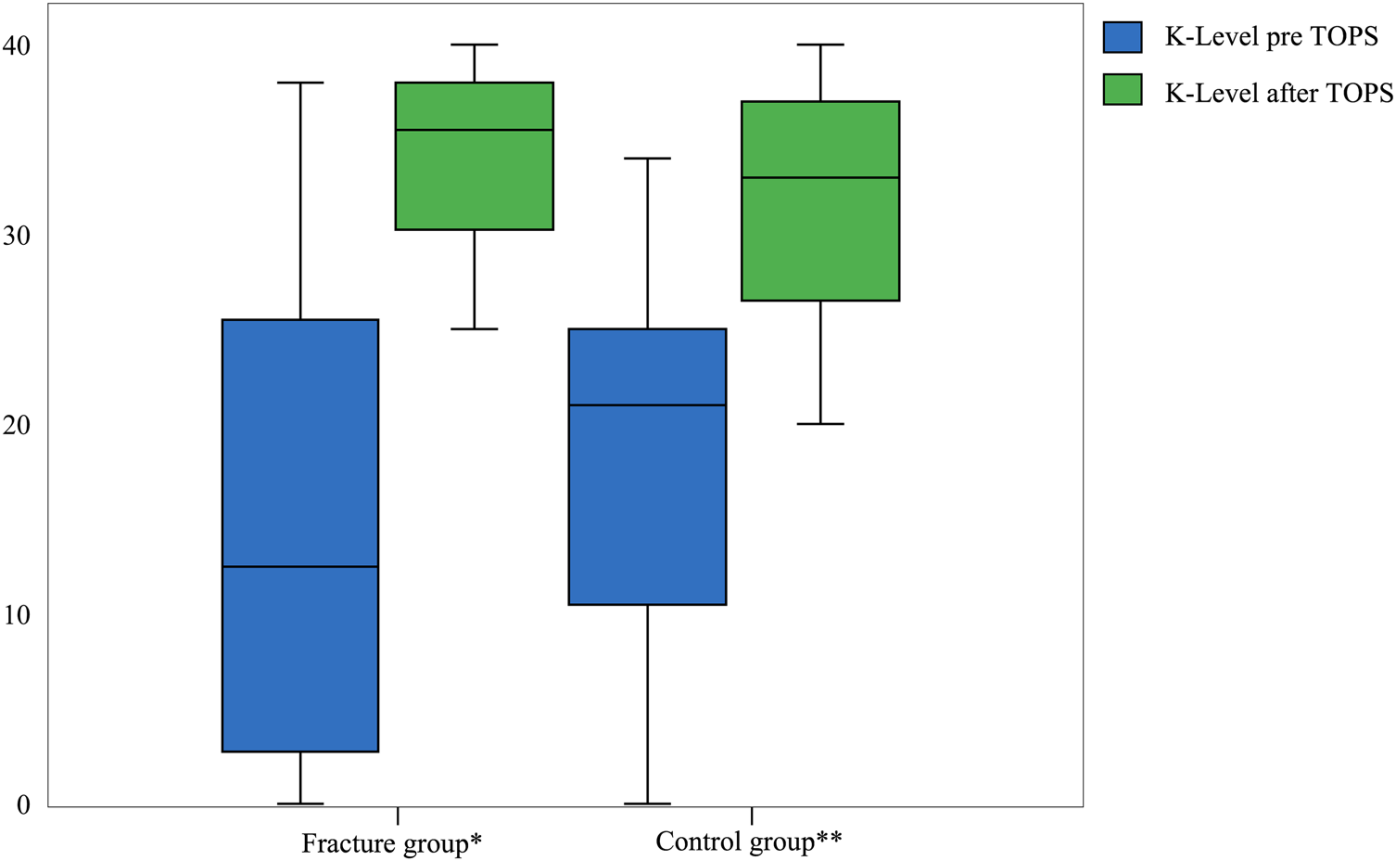
Graphical illustration of the comparison of the PMQ before and after TOPS for the fracture and control group

**Fig. 3:**
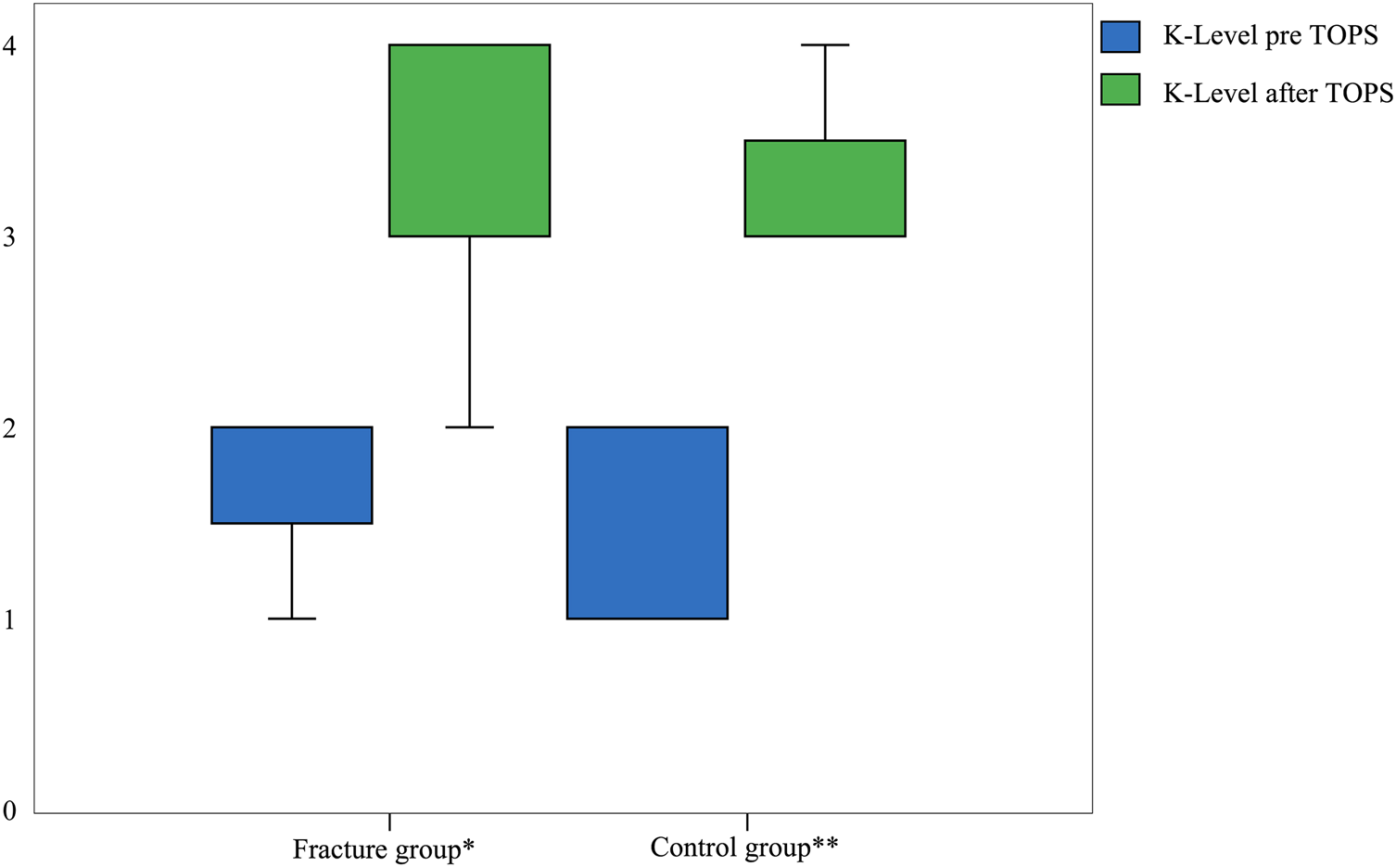
Graphical illustration of the comparison of the K-level before and after TOPS for the fracture and control group

There was no significant difference for PMQ and K-level between the fracture and control group at follow-up times. In contrary, the fracture and control group showed a highly significant difference between PMQ and K-level before and after TOPS supply. Detailed results are shown in Figs. 2 and 3.

**Fig. 4:**
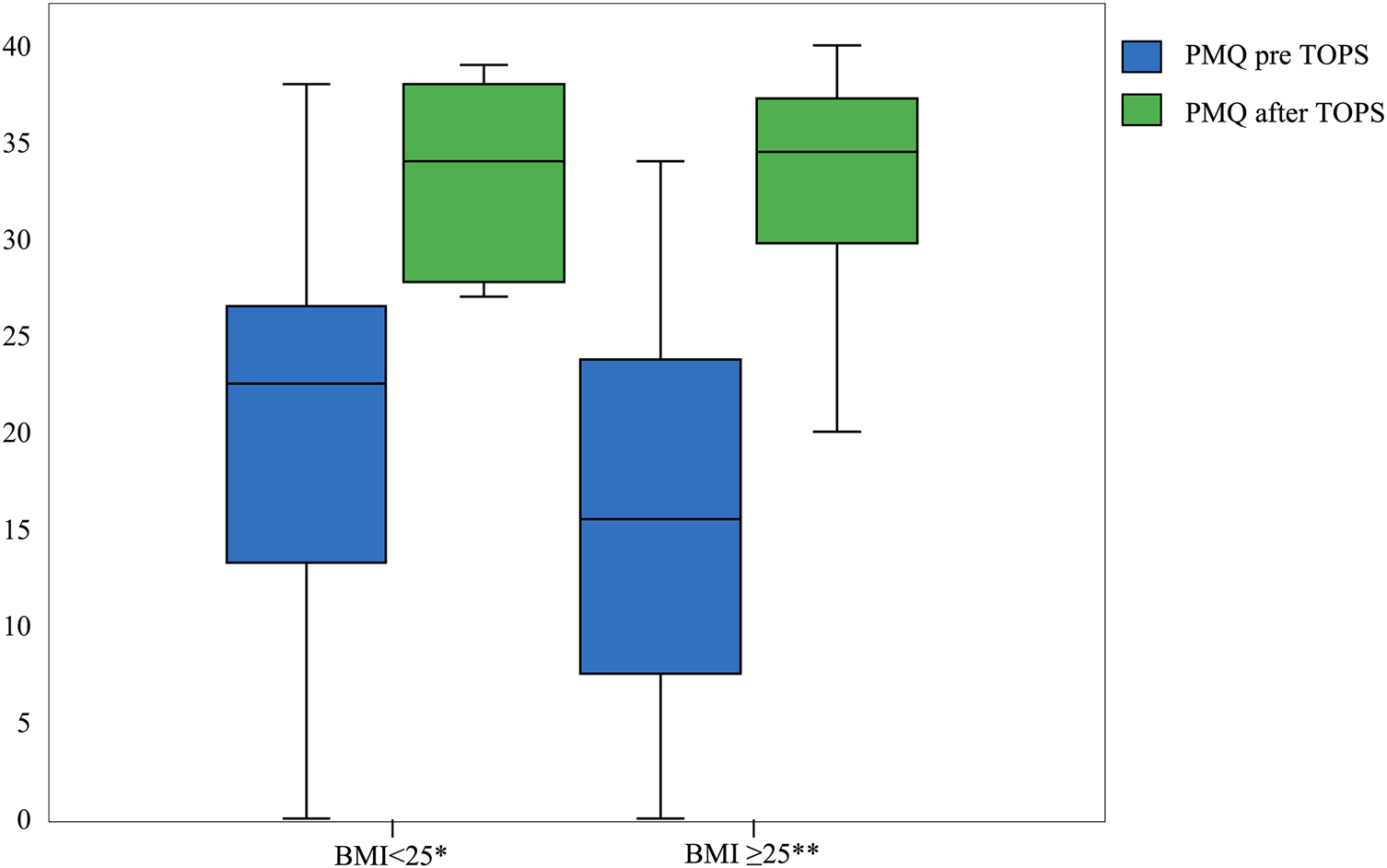
Graphical illustration of the comparison of the PMQ before and after TOPS for the subgroup analysis (BMI)

**Fig. 5:**
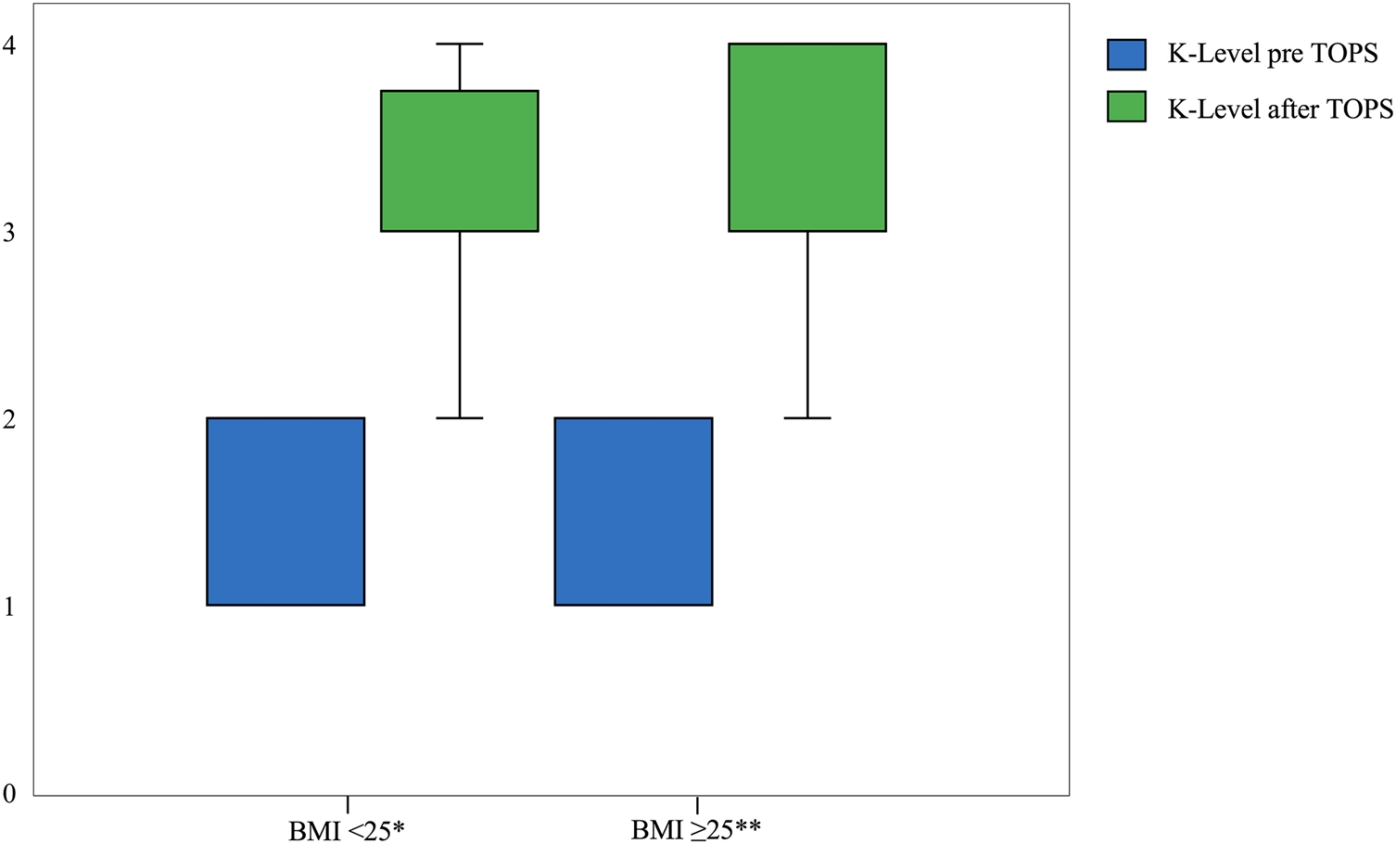
Graphical illustration of the comparison of the K-level before and after TOPS for the subgroup analysis (BMI)

The subgroup analysis (BMI < 25 kg/m^2^ vs. BMI ≥ 25 kg/m^2^) shows for the age at amputation (*p* = 0.002^b^) a significant difference between these two groups. Also, the comparison of the PMQ before and after TOPS showed significant differences. The comparison of the other parameters showed no significant difference. Detailed information is shown in Table 3 as well as Figs. 4 and 5.

**Table 3:** Demographic data of the subgroups according to the BMI

	BMI < 25 kg/m^2^ (*n* = 12)	BMI ≥ 25 kg/m^2^ (*n* = 22)	*p* value
BMI [kg/m^2^] mean ± SD (95%-CI)	22.2 ± 2.1 ( (20.8–23.5)	29.1 ± 3.0 (27.7–30.4)	0.002^b^
Sex, *n* (%)			0.7^a^
Male	8 (66.7)	17 (77.3)	
Female	4 (33.3)	5 (22.7)	
Side, *n* (%)			0.049^d^
Left	6 (50)	15 (68.2)	
Right	4 (33.3)	7 (31.8)	
Bilateral	2 (16.7)	0	
Reason for amputation, *n* (%)			0.1^d^
Trauma	10 (83.3)	13 (59.1)	
Tumor	0 (0)	1 (4.5)	
Vascular disease	1 (8.3)	0	
Iatrogenic complications	1 (8.3)	8 (36.4)	
Fracture, *n* (%)			0.7^a^
Yes	6 (50)	9 (40.9)	
No	6 (50)	13 (59.1)	
Age [years] mean ± SD (95%-CI)	46.1 ± 9.2 (40.2–52.0)	50.2 ± 9.8 (45.9–54.5)	0.2^e^
Age at amputation [years] mean ± SD (95%-CI)	25.8 ± 11.2 (18.7–33.0)	36.9 ± 12.4 (31.4–42.3)	< 0.001^b^
Months amputation to TOPS mean ± SD (95%-CI)	204.7 ± 171.4 (95.6–313.6)	119.7 ± 97.9 (76.4–163.2)	0.1^e^
Use of prostheses [hours] mean ± SD (95%-CI)	11.9 ± 4.9 (8.8–15.0)	13.2 ± 3.5 (11.7–14.8)	0.6^b^
ASA Score mean ± SD (95%-CI)	1.9 ± 0.3 (1.7–2.1)	2.1 ± 0.4 (1.9–2.2)^**^	0.5^d^
CCI [%] mean ± SD (95%-CI)	97.2 ± 1.0 (96.5–97.8)	92.8 ± 10.7 (88.0–97.5)^**^	0.1^b^

### Novel Classification System–Hannover Classification of intra- and postoperative periprosthetic Fractures (HCiopF, HCpopF) and treatment algorithm

This rehabilitation system (TOPS) with its intramedullary implant which passes through the skin by two times surgery is new and still used only in a few centers worldwide. Therefore, treatment recommendations for such as cases are spares. Of particular note and in contrast to other classifications [22, 23], we recommend to not remove or exchange the implant (Endo-Fixstem) even if it is assembly. We assume that no relevant implant loosening occurs in the context of postoperative periprosthetic fractures due to the special surface structure (so-called tripods) of the endo-fixed stem and the circumferential osseointegration. If loosening does occur, the recommendation would still be just to perform implant-retaining osteosynthesis and whenever possible, not to remove the implant.

The novel classification system consists of three categories A–C (Fig. 8). Category A represents the most simple and normally stable fracture morphology, such as a longitudinal split of the cortical bone and affecting the distal femur diaphysis. Oftentimes, conservative treatment is recommended for such cases of type A. Type C represents the most difficult form of periprosthetic fractures, mostly unstable and affecting the metaphysis of the femur as well as the trochanteric region and requiring implant-retaining osteosynthesis. Type B contains fracture types in between types A and C. Detailed information about the classification and its treatments is shown in Tables 4 and 5.

**Table 4:** Hannover Classification of intraoperative periprosthetic Fractures (HCiopF) as part of TOPS treatment and the treatment algorithm to the Hannover Classification of intraoperative periprosthetic Fractures (HCiopF)

Type	Description	Subtypes	Treatment
A	Fractures of the distal femoral bone	1: Simple cortical perforation* 2: Undisplaced linear crack 3: n/a	Local bone grafting, Protected weight bearing
B	Fractures affecting the proximal diaphysis	1: Simple cortical perforation 2: Undisplaced linear crack 3: Displaced or unstable fracture	1. Local bone grafting, Protected weight bearing 2. Local bone grafting, Protected weight bearing, cerclage wire 3. Internal fixation with cerclage wire, plate osteosynthesis***
C	Fractures affecting proximal metaphysis and trochanteric region	1: Simple cortical perforation 2: Undisplaced linear crack 3: Displaced or unstable fracture	1. Local bone grafting, Protected weight bearing 2. Local bone grafting, Protected weight bearing, cerclage wire 3. Internal fixation with cerclage wire, plate osteosynthesis***

*e.g., by intraoperative drilling; ***DHS, DCS plate, LISS plate, Condylplate, or individual implant

**Table 5:** Hannover Classification of postoperative periprosthetic Fractures (HCpopF)as part of TOPS treatment as well as the treatment algorithm to the Hannover Classification of postoperative periprosthetic Fractures (HCpopF)

Type	Description	Subtypes	Treatment
A	Fractures affecting the distal area of the femoral bone	1: Undisplaced linear crack 2: Displaced or unstable fracture	1. Local bone grafting, Protected weight bearing 2. Protected weight bearing, cerclage wire or open reduction and internal fixation with locking plate***
B	Fractures affecting the proximal diaphysis	1: Femoral stem well fixed 2: Femoral stem loose 3: Femoral stem loose with severe loss of bone stock	1. Local bone grafting Protected weight bearing 2. Open reduction and internal fixation with locking plate 3. Open reduction and internal fixation with locking plate***
C	Fractures affecting proximal metaphysis and trochanteric region	1. Trochanter minor (*undisplaced, **displaced) 2. Trochanter major (displaced > or < two centimeters) 3. Inter-/subtrochanteric	1. * Local bone grafting Protected weight bearing 1. ** Protected weight bearing, internal fixation 2. < 2 cm displaced: nonoperative 2. > 2 cm displaced: open reduction and internal fixation 3. Open reduction and internal fixation with locking plate*** or proximal femoral arthroplasty

***DHS, DCS-Plate, LISS-Plate, Condylplate or individual Implant

## Discussion

The most important finding of this study was that periprosthetic fractures following TOPS did not negatively affect outcomes.

For both the fracture and control group, a significant increase of the PMQ and K-level was observed before and after TOPS treatment. Subgroup analysis regarding BMI showed a significantly higher increase of the PMQ before and after TOPS for the group BMI ≥ 25 than for the group BMI < 25, regardless of a periprosthetic fracture. Subgroup analysis of the K-level also changed significantly for both groups before and after TOPS treatment. There was no significant improvement in the rehabilitation results for the K-level in favor of the overweight patients compared to the normal weight patients.

According to the findings of Hoellwarth et al. [12], our results confirm that periprosthetic fractures after TOPS treatment do not necessarily have a negative impact onto rehabilitation success. To the present time, all fracture fixations have been rehabilitated satisfactorily.

**Fig. 6:**
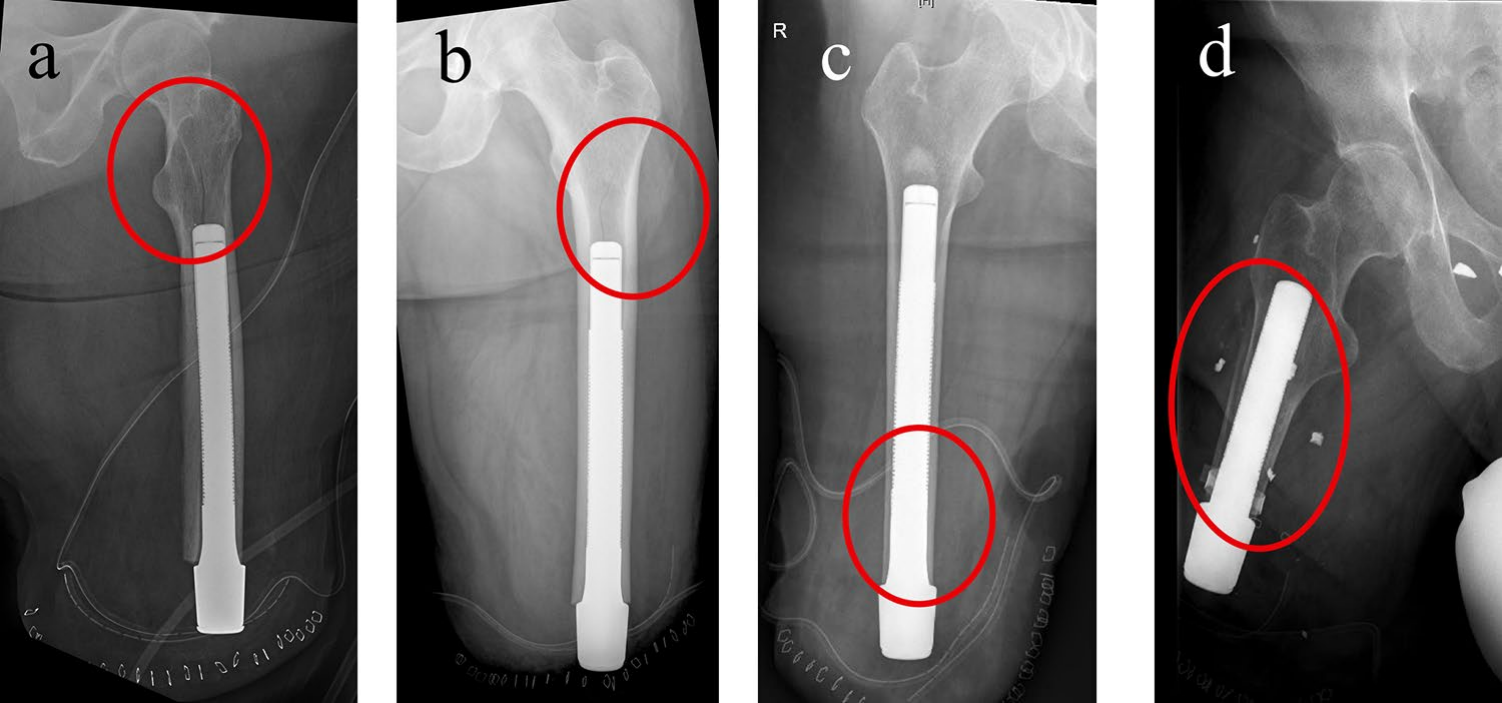
**a**–**d** Intraoperative periprosthetic fractures after implantation of the Endo-Fixstem; **c** the split fracture is covered by the implant; Only **d** needs an intraoperative fixation by cerclage cable

**Fig. 7:**
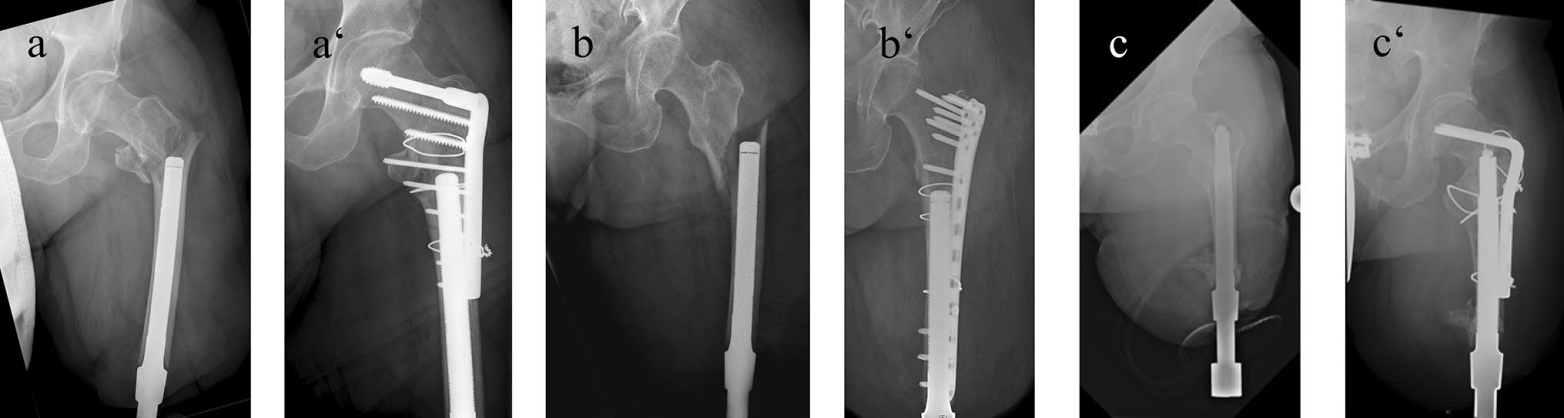
**a**–**c** X-rays with postoperative periprosthetic fractures before and after open reduction and internal fixation with plates and cerclage cable

Surgical treatment of periprosthetic fractures after TOPS treatment was performed individually, according to their fracture morphology (Figs. 6, 7). The Endo-Fixstem never had to be exchanged or removed. In order to ensure a successful TOPS treatment, it is necessary to achieve a high primary stability in the first surgical step with a long press-fit anchorage [24]. For this purpose, the entire remaining diaphysis of the residual bone is mostly used for implant anchoring, resulting in placement of the tip of the Endo-Fixstem at the level of the lesser trochanter. In the context of a fall with a consecutive periprosthetic fracture, fractures are frequently encountered in the intertrochanteric and femoral neck area.

**Fig. 8:**
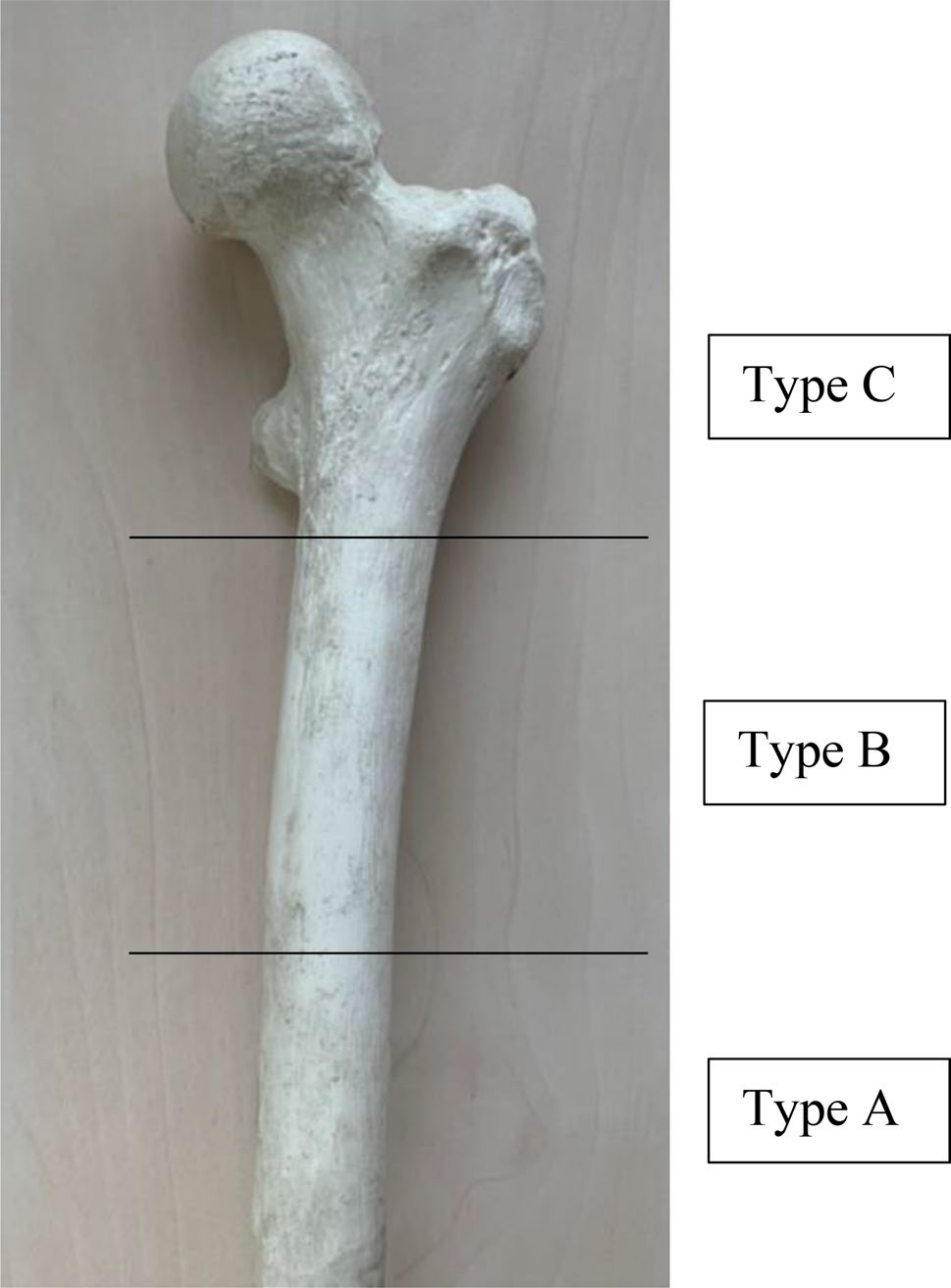
Description of the level for intra- and postoperative periprosthetic fracture of the residual femur, fracture localization for classification

Based on the Vancouver Classification [22, 23] for intra- and postoperative periprosthetic fractures of total hip arthroplasty (THA), a novel Hannover Classification System and treatment recommendation (Fig. 8, Tables 4, 5) of periprosthetic fractures after TOPS treatment was derived.

In contrast to the Vancouver Classification, it can be assumed that for postoperative periprosthetic fractures the Endo-Fixstem will not be loosen in most cases. Therefore, a change to a longer Endo-Fixstem with a hole containing a Femoral Lag Screw would not be possible or only with great effort. This is due to the fact that a very tight connection is formed between the surface structure (tripods) of the Endo-Fixstem and the bone [24–26]. This aspect could result in intraoperative fractures (Fig. 6). In postoperative periprosthetic fractures, it increases the degree of difficulty of implant-retaining osteosynthesis treatment, but could be a positive factor for rehabilitation after a periprosthetic fracture, since "only" the fracture would have to heal if the implant was firmly anchored. Tables 4 and 5 depict the classification and treatment algorithms.

Furthermore, it appears that overweight patients benefit even more from TOPS treatment than normal weight patients. Nevertheless, the BMI is regarded in the literature as a risk factor for complications such as periprosthetic fractures after arthroplasty surgeries [27]. In contrast to our results, both Hoffmann et al. and Canton et al. report a mean BMI of 32.4 kg/m^2^ [28], and BMI > 30 kg/m^2^ [29], respectively, considering this to be a predictor for a periprosthetic fracture.

Besides obesity, medical comorbidities such as cardiac and neurologic pathologies can contribute to ambulation instability with consecutively higher risk of fall and need to be considered as additional risk factors [29]. Other authors report that increased age and female gender may be a predictor of increased risk of periprosthetic fracture [29–35]. Again, this could not be confirmed in our cohort, which mainly consisted of younger (mean age 48.7 years) and predominantly male (73.5%) patients. Derived from this, the incidence of periprosthetic fractures after TOPS could be attributed to an increased level of activity and an increased risk disposition [36].

Our study was conducted with a small cohort, but our results showed a significant improvement in mobility after TOPS treatment regardless of periprosthetic fracture. In consideration of the results of the subgroup analysis, future studies will be necessary to clarify whether the BMI should be considered a predictor for the rehabilitation success when using TOPS to major limb loss of the lower extremity. These aspects emphasize the importance of TOPS as a valid rehabilitation alternative for major limb loss.

The satisfactory results from the fracture group provide evidence for our chosen osteosynthesis procedures, so the derived classification and treatment algorithms could be included in the planning of the treatment of periprosthetic fractures according to TOPS in the future.

### Limitations

A limiting factor is the low number of this cohort. This can be explained by still a rare use of TOPS as a rehabilitation alternative for transfemoral amputees both in Germany and worldwide. Since 2010, in Germany 140 patients have been treated with TOPS. In relation to this number of TOPS patients, the number of cases in the fracture group represents approximately 10% of all patients treated in Germany. However, it should be noted that five cases were intraoperative fractures that did not require further surgical intervention and could be treated sufficiently by a conservative procedure. In addition, widely spread time periods both between amputation and TOPS treatment, and between TOPS treatment and periprosthetic fractures were observed. It is unclear what bone quality at the time of implantation of the Endo-Fixstem TOPS user had, as an osteopenia bone structure according to time between amputation and TOPS treatment, can increase the risk of intra- and postoperative periprosthetic fractures. The limiting factors occurred involuntarily and randomly, so that these aspects could not direct influence the study design as a bias.

## Conclusion

Periprosthetic fractures do not necessarily worsen outcomes of TOPS treatment. Proper classification, standardized appropriate treatment strategies according to fracture morphology are paramount for reliably good outcomes. We recommend to not remove or exchange the implant (Endo-Fixstem) even if it is assembly. Higher BMI did not have an impact onto rehabilitation success after TOPS to major limb loss of the lower extremity.

## Abbreviations

ASA: American Society of Anesthesiologists
BMI: Body mass index
CCI: Charlson Comorbidity Index
EEP: Endo-exo-prosthesis
DCS: Dynamic condyle screw
DHS: Dynamic hip screw
HCiopF: Hannover classification of intraoperative periprosthetic fractures
HCpopF: Hannover classification of postoperative periprosthetic fractures
HCFA: US Health Care Financing Administration
HRQoL: Health-related quality of life
LISS: Less invasive stabilization system
PMQ: Prosthesis Mobility Questionnaire
TOPS: Transcutaneous osseointegrated prosthetic systems
e.g.: Exempli gratia
